# An Eye on Efficiency: A Quality Improvement Project to Increase Ophthalmology Equipment in the Accident and Emergency Department

**DOI:** 10.7759/cureus.76032

**Published:** 2024-12-19

**Authors:** Jacques le Roux, Ayushi Gupta, Jaykumaran Govender

**Affiliations:** 1 Emergency Medicine, Whittington Health NHS Trust, London, GBR; 2 Ophthalmology, Royal Free Hospital, London, GBR

**Keywords:** emergency department, emergency medicine, ophthalmology assessment, ophthalmoscope use, quality improvement project

## Abstract

Introduction

Ophthalmology presentations contribute significantly to Accident and Emergency (A&E) attendance. The provision of safe care depends on clinical skills and appropriate equipment. This quality improvement project aimed to increase the availability of ophthalmoscopes and Snellen charts required for a basic ophthalmological assessment in an A&E department in North London.

Methods

The time taken for 23 permanent staff to retrieve a working ophthalmoscope and 9 permanent staff to find a Snellen chart, respectively, were recorded in different areas of the department. Following this, broken ophthalmoscopes were replaced, and additional ophthalmoscopes and Snellen charts were installed. The task was repeated post-intervention.

Results

Pre-intervention, there was 1 ophthalmoscope per 4.9 cubicles which increased to 1 per 1.7 cubicles post-intervention. The number of ophthalmoscopes in paediatric A&E and the number of mobile ophthalmoscopes increased the most. The median time taken to find a working ophthalmoscope anywhere in the department was 53 seconds pre-intervention and 19 seconds post-intervention. The median time taken to find a Snellen chart was 90 seconds pre-intervention and 32 seconds post intervention.

Conclusions

This quality improvement project successfully increased the number of ophthalmoscopes available in the department to meet local targets in most areas of A&E except for the major injuries and resuscitation areas. The time taken to find a working ophthalmoscope and Snellen chart was significantly reduced. Based on the number of patients presenting to our A&E with eye complaints over a one-year period between 01/07/23 and 01/07/24, these interventions will save approximately 13.6 clinician working hours searching for an ophthalmoscope and 15.4 clinician working hours searching for a Snellen chart.

## Introduction

Ophthalmology presentations account for a significant burden of Accident and Emergency (A&E) attendances in the United Kingdom (UK), with the Royal College of Ophthalmologists estimating these to account for 1.46-6% of presentations to a regular A&E (not a designated Eye Casualty) [1]. Patients present with a range of complaints from non-urgent to sight-threatening conditions. The provision of safe triage and appropriate care for these patients depends on effective and timely diagnosis. Despite the need for high-quality examination, A&E departments often lack available working equipment and doctors report low confidence in using ophthalmoscopes [2,3]. 

The number of patients presenting to emergency departments and waiting times across the UK are increasing [4,5]. In these busy environments, clinicians are required to complete preliminary assessments with minimal equipment and knowledge [2,3]. Essential equipment for an ophthalmological examination includes visual acuity charts and ophthalmoscopes. Lack of access to these will delay diagnosis, resulting in unnecessary referrals to speciality services, and can pose a patient safety concern where delayed care can have sight-threatening consequences [6-8]. 

 This quality improvement project aimed to evaluate and improve the availability of ophthalmological equipment within our A&E department. By reviewing current resources, identifying gaps and implementing targeted interventions, we aim to provide better care for our patients. 

## Materials and methods

This project was conducted in the A&E Department of Whittington Hospital, a district general hospital in London, United Kingdom. The department is a 46-bed unit with additional seating areas, and in 2023 provided care to 79,589 patients. Of these, 49,807 (63%) were triaged to urgent care or general practitioners. There are 67 permanent staff. The project was carried out over a six-month period, beginning with a baseline assessment of available ophthalmological equipment and concluding with a post-intervention evaluation of equipment availability and accessibility. 

To establish the baseline, the total number of working ophthalmoscopes and Snellen charts in the department was counted. The counts were conducted systematically, with each clinical area assessed independently by two investigators to ensure accuracy. The areas assessed included urgent care, paediatrics, majors, resuscitation, and the rapid assessment and treatment area. Mobile units were also included in the count. These numbers were then compared to local departmental guidelines produced by service managers and consultants (Appendix 1), which specify the minimum number of ophthalmoscopes. 

Functionality and accessibility of equipment were assessed by tasking clinicians with locating a working ophthalmoscope or Snellen chart. A total of 23 clinicians were asked to find a working ophthalmoscope, and 9 clinicians were asked to locate a Snellen chart. The time taken to complete each task was recorded using a stopwatch, with timing initiated at the clinician’s starting point in their respective clinical area and stopped when they returned with the required equipment. The Whittington Hospital A&E is a small department with a variable number of staff available each day, therefore this exercise was conducted over a period of one week at multiple times of the day to simulate realistic conditions and account for variations in shift business. The difference in the number of participants used for the ophthalmoscope and Snellen chart tasks reflects the availability of staff to partake in each activity.

Following this, the number of fixed and mobile ophthalmoscopes and Snellen charts was increased in line with departmental guidelines. A further 23 clinicians were asked to locate and return with a working ophthalmoscope, and 9 clinicians repeated the task for Snellen charts. The process of timing both groups were conducted in the same manner, ensuring consistency between the pre- and post-intervention assessments. Additionally, the total number of working ophthalmoscopes and Snellen charts in the department was recounted to determine the overall increase in availability. This post-intervention count was performed by the same investigators. The data was analysed to calculate a change in the number of available equipment and the time taken to locate them before and after the intervention. Statistical analysis of the difference in time taken was performed with GraphPad Prism version 10.2.3 (Dotmatics, Boston, MA) using the Kruskall-Wallis method to compare non-parametric values. A p-level of 0.05 or less was considered significant.

## Results

The total number of ophthalmoscopes in the department increased from 10 to 29; pre-intervention, there was 1 ophthalmoscope per 4.9 cubicles which increased to 1 per 1.7 cubicles post-intervention (Table 1). There was an increase in fixed ophthalmoscopes in the urgent care, paediatrics and rapid assessment and treatment areas (Table 2). There was also an increase in the number of loose ophthalmoscopes (Table 2). The number of Snellen charts in the department increased from 1 to 11 (7 in urgent care, 2 in paediatrics and 2 in majors). 

**Table 1:** Total number of ophthalmoscopes in the department

Parameter	Number	Ratio
Pre-intervention	10 for 46 cubicles	1 per 4.6 cubicles
Post-intervention	29 for 46 cubicles	1 per 1.6 cubicles

**Table 2:** Number of ophthalmoscopes available in each area

Location	Pre-intervention	Post-intervention	Locally approved 'gold standard'
Urgent care	3/9 cubicles	5/9 cubicles	4
Paediatrics	1/7 cubicles	7/7 cubicles	5
Majors	0/21 cubicles	0/21 cubicles	11
Resus	0/4 cubicles	0/4 cubicles	4
Rapid assessment and treatment	3/5 cubicles	4/5 cubicles	5
Loose	3	12	4

**Figure 1:**
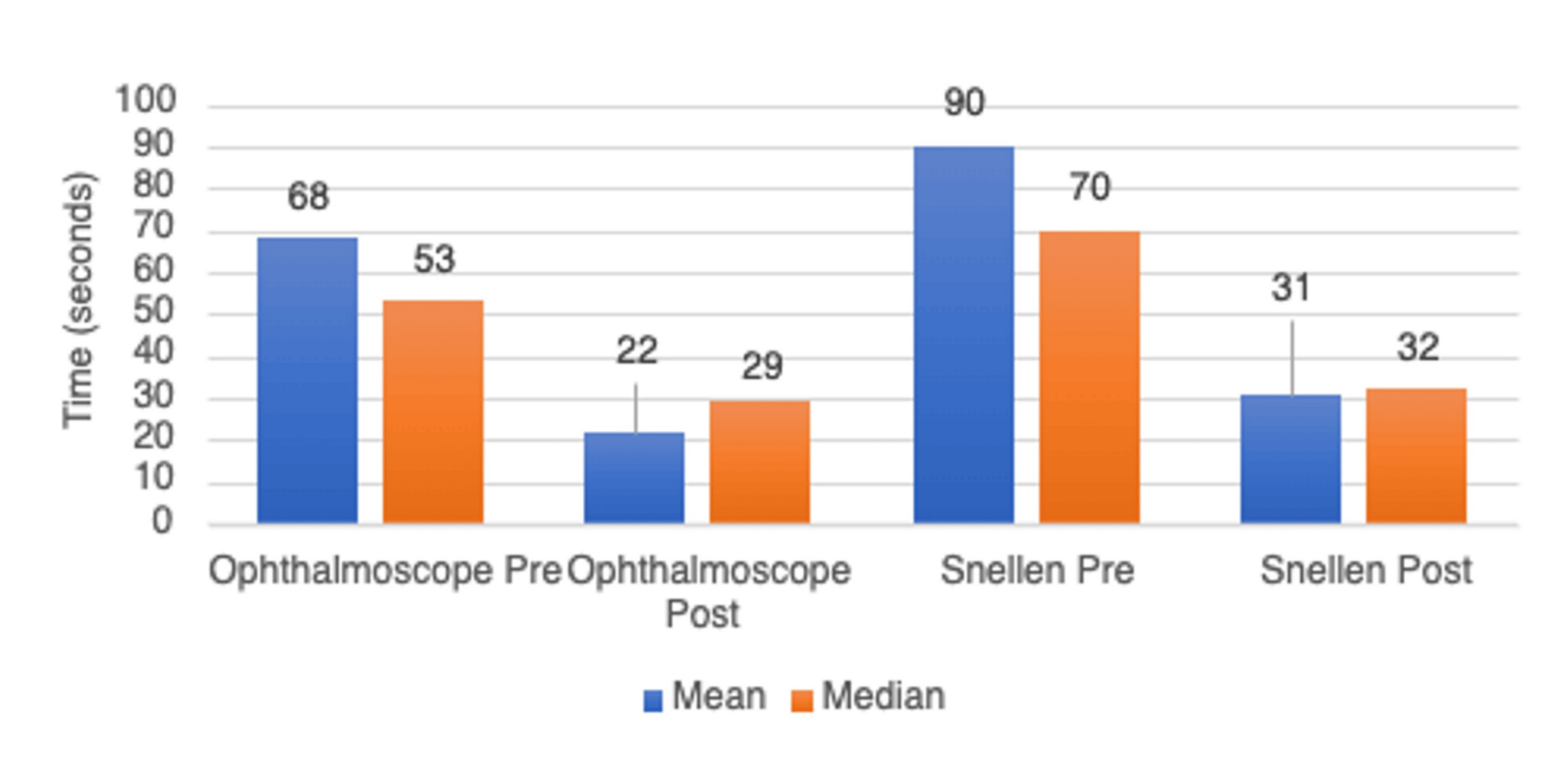
Average time taken (seconds) to find a working ophthalmoscope or Snellen chart

The median time taken to find a working ophthalmoscope in the department was 53 seconds pre-intervention (range from 24 seconds to 2 minutes 10 seconds) and 19 seconds post-intervention (range from 2 seconds to 1 minute 11 seconds) (Figure 1). This corresponds to a statistically significant reduction of 34 seconds, or 45.28% (p<0.0001). 

The median time taken to find a Snellen chart was 90 seconds pre-intervention (range from 4 seconds to 343 seconds) and 32 seconds post-intervention (range 6 seconds to 52 seconds) (Figure 1). This corresponds to a statistically significant median reduction of 38 seconds, or 64.44% (p=0.02). 

## Discussion

This quality improvement project demonstrates an increase in the availability of basic ophthalmological equipment in the A&E department. The increased number of ophthalmoscopes and Snellen charts led to a significant reduction in the time taken for clinicians to locate equipment. Although the Royal College of Ophthalmologists emphasises the importance of ophthalmic equipment in the A&E, there are no national guidelines for an appropriate number of ophthalmoscopes for an Emergency Department [9]. However, a local guideline created by the Emergency Department’s management has been provided in Appendix 1. The department now meets or has reached near to local targets in most areas of A&E except for major injury and resuscitation areas. These two areas are covered by mobile units which can be moved to the necessary bay. Looking for a working ophthalmoscope is an example of a functional bottleneck, which reduces flow in the department [10]. Reducing time spent looking for functional equipment improves the clinician experience and reduces patient length of stay in the A&E [10-12]. Furthermore, thorough examination reduces the risk of misdiagnoses or delayed diagnoses. These factors all improve patient care and flow. Reducing waiting times also leads to a reduction in the number of patients who leave before being seen or leave against medical advice [13]. 

The Whittington A&E department saw 1,445 patients presenting with eye complaints between the 1st of July 2023 and the 1st of July 2024. Therefore, over a one-year period, our interventions can be expected to save approximately 13.6 clinician-working hours spent looking for a working ophthalmoscope and 15.4 clinician-working hours spent looking for Snellen charts in our department. Furthermore, this figure is likely to be an underestimate as many other patients will also require the use of the ophthalmoscope and otoscope set, such as those presenting with headache, dizziness, trauma and severe hypertension. There were 23.6 million visits to NHS A&E departments in 2023 [14]. If similar interventions were implemented across the NHS, where 30 seconds are saved each time a patient requires an ophthalmological assessment, and 6% of presentations require such assessment [1], more than 700,000 clinician hours would be saved per year. This time saved could contribute to reducing waiting times for patients [10,15]. Reducing the need to search for equipment also contributes positively to the clinician's workplace experience. Ophthalmoscopes and Snellen charts are just a few of the many different assessment tools used in A&E daily practice; this project shows the power of adequately equipping an A&E department. 

This study had several limitations. It was a small study conducted within a single A&E in a district general hospital without onsite ophthalmology support. This limits its generalisability to other departments with different resources or speciality pathways. Additionally, our project focused on the availability of physical resources without assessing the clinician's use of the equipment. Finally, this study did not determine the retention of equipment over time, particularly as mobile ophthalmoscopes may be at risk of damage or loss. We recognise the need for regular equipment checks. 

Future work should focus on increasing the period studied and expanding the project into other departments to generalise these findings. There is ongoing work in our A&E to upskill clinicians in fundoscopy, improving confidence and diagnostic accuracy. 

## Conclusions

Our quality improvement project successfully increased the availability of essential ophthalmological equipment in our A&E and reduced the time taken to find essential equipment for an ophthalmology assessment. This represents a huge improvement in time saved looking for equipment over a one-year period, which reduces waiting times and improves patient experience. Further work is needed to identify the long-term impact of this study and confirm its national impact, carry out work to determine the minimum number of ophthalmoscopes according to department size for cost-effectiveness, and ensure clinicians are trained in the use of specialist equipment. 

## Appendices

Appendix 3

Table 3 shows the local guidelines for the minimum number of ophthalmoscopes in the A&E.

**Table 3:** Local guidelines for the minimum number of ophthalmoscopes in A&E

Location	Locally approved 'gold standard'	Minimum recommendation
Majors	11, 1 mobile	11, 1 mobile
Green majors	1 mobile	1 mobile
Resus	4	1 mobile
Urgent care	4, 1 mobile	4, 1 mobile
Rapid assessment and treatment	5	1 mobile
Paediatrics	5, 2 mobile	5, 2 mobile
